# High-Performance Anti-freezing Flexible Zn-MnO_2_ Battery Based on Polyacrylamide/Graphene Oxide/Ethylene Glycol Gel Electrolyte

**DOI:** 10.3389/fchem.2020.00603

**Published:** 2020-07-31

**Authors:** Yuhui Quan, Minfeng Chen, Weijun Zhou, Qinghua Tian, Jizhang Chen

**Affiliations:** ^1^College of Materials Science and Engineering, Nanjing Forestry University, Nanjing, China; ^2^Department of Chemistry, School of Sciences, Zhejiang Sci-Tech University, Hangzhou, China; ^3^Co-innovation Center of Efficient Processing and Utilization of Forest Resources, Nanjing Forestry University, Nanjing, China

**Keywords:** hydrogel electrolytes, environmental adaptive, Zn-ion batteries, manganese dioxides, aqueous energy storage

## Abstract

It remains a great challenge for aqueous zinc-ion batteries to work at subzero temperatures, since the water in aqueous electrolytes would freeze and inhibit the transportation of electrolyte ions, inevitably leading to performance deterioration. In this work, we propose an anti-freezing gel electrolyte that contains polyacrylamide, graphene oxide, and ethylene glycol. The graphene oxide can not only enhance the mechanical properties of gel electrolyte but also help construct a three-dimensional macroporous network that facilitates ionic transport, while the ethylene glycol can improve freezing resistance. Due to the synergistic effect, the gel electrolyte exhibits high ionic conductivity (e.g., 14.9 mS cm^−1^ at −20 °C) and good mechanical properties in comparison with neat polyacrylamide gel electrolyte. Benefiting from that, the assembled flexible quasi-solid-state Zn-MnO_2_ battery exhibits good electrochemical durability and superior tolerance to extreme working conditions. This work provides new perspectives to develop flexible electrochemical energy storage devices with great environmental adaptability.

## Introduction

Currently, wearable electronics remain a leading trend of next-generation consumer electronics (Dong and Wang, 2019). Although lithium-ion batteries (LIBs) with high energy density have been widely used in the fields of portable electronics and electric vehicles, they are involved with toxic and flammable organic electrolytes, thus restricting their applications in wearable electronics. Among numerous candidates for wearable electronics, rechargeable aqueous zinc-ion batteries (AZIBs) stand out owing to their high safety, low manufacturing cost, and environmental friendliness, as well as the abundance of zinc sources (Ming et al., 2019). Besides, aqueous electrolytes offer a series of advantages (including high ionic conductivity of ~100 mS cm^−1^) over organic electrolytes (Deng et al., 2020; Xie et al., 2020). Nevertheless, liquid electrolytes would leak from AZIBs under strain, which would limit the flexibility of AZIBs. Moreover, AZIBs usually suffer from rapid capacity fading and poor cycling stability, due to the irreversible side reactions on both cathodes and anodes and the dissolution of cathode materials into electrolytes (Li et al., 2019; Tang B. Y. et al., 2019; Huang et al., 2020). To address these issues, flexible gel electrolytes emerge as a good choice (Huang et al., 2019; Lei et al., 2020). Compared to liquid electrolytes, gel electrolytes have the following advantages (Zhang et al., 2018; Wu et al., 2020). Firstly, the leakage issue of liquid electrolytes does not exist in gel electrolytes, and the latter can provide great physical flexibility. Secondly, gel electrolytes can be adsorbed onto the growth points of zinc dendrites and therefore inhibit the further growth of zinc dendrites. Thirdly, gel electrolytes with limited free water can effectively suppress the dissolution of active materials. Last but not least, separators are not required when gel electrolytes are used.

Polyvinyl alcohol (PVA)-based gel electrolytes have been widely used for aqueous flexible electrochemical energy storage devices (Chen et al., 2018; Chen M. F. et al., 2019; Wang et al., 2020). For example, Lu et al. used a PVA/ZnCl_2_/MnSO_4_ gel electrolyte to construct flexible Zn/MnO_2_ battery, which can retain more than 77.7% and 61.5% of its initial capacity after 300 and 1,000 cycles, respectively (Zeng et al., 2017). However, the gel electrolytes based on PVA have the disadvantages of low elasticity, low ionic conductivity, and poor mechanical strength, due to the absence of cross-linking points among PVA molecular chains (Huang et al., 2019; Li et al., 2019). Besides, the ${\text{SO}}_{4}^{2-}$ ions in commonly used ZnSO_4_ electrolytes for AZIBs would easily precipitate PVA (Cao et al., 2019). Beyond PVA, polyacrylamide (PAM) (Li et al., 2018b; Wang Z. et al., 2018), gelatin (Li et al., 2018b; Zhao et al., 2019), xanthan gum (Zhang et al., 2018), sodium polyacrylate (Huang Y. et al., 2018), and fumed silica (Murali and Samuel, 2019) were also reported to construct gel electrolytes for AZIBs. Among them, PAM gel electrolytes are very promising, benefiting from the structural merits of PAM (Huang et al., 2017; Song et al., 2018). On the one hand, nearly all the PAM chain segments are connected by cross-linking, contributing to better mechanical properties than PVA counterparts. On the other hand, the hydrophilic groups (especially –CONH_2_ amide groups) can trap sufficient Zn^2+^-containing aqueous solution within the PAM porous framework, facilitating ionic mobility. However, it is difficult to realize great mechanical properties and very high ionic conductivity for neat PAM gel electrolytes (Tran et al., 2019). To overcome this obstacle, nanocellulose (Wang D. et al., 2018) and gelatin (Li et al., 2018a) were added to the PAM matrix, and great electrochemical performances were obtained for the assembled flexible batteries. In this study, we use graphene oxide (GO) as the additive to improve the properties of PAM gel electrolytes. It is worth mentioning that GO has proven to be rather effective in enhancing ionic conductivity of gel polymer electrolytes (Yang et al., 2013). With abundant oxygen-containing functional groups, GO is also anticipated to form numerous hydrogen bonds with PAM, thus reinforcing the PAM network.

As a result of a large amount of water in the gel electrolytes, the batteries with conventional gel electrolytes definitely show poor performances in the low-temperature environments, due to that the gel electrolytes would freeze at subzero temperatures (Mo et al., 2019). However, energy-storage devices are required to work in low-temperature environments, since it is cold in winter in many regions around the world. It has been reported that adding cryoprotectants such as glycerol and ethylene glycol (EG) into gel electrolytes can realize good anti-freezing capabilities (Mo et al., 2019; Zhou et al., 2020). In our previous report, we developed a borax-cross-linked PVA/glycerol gel electrolyte, in which glycerol can interact with PVA chains strongly, thus effectively preventing the formation of ice crystals within the whole gel network (Chen et al., 2020). Even at −20°C, it still shows a high ionic conductivity of 15.9 mS cm^−1^ and great mechanical properties. Herein, we demonstrate the construction of the PAM/GO/EG gel electrolyte, aiming to improve ionic conductivity, mechanical properties, and anti-freezing capability of PAM gel electrolytes by utilizing the synergistic effect of EG and GO. Meanwhile, we choose MnO_2_ as the cathode material for Zn^2+^ ion storage in this study, as MnO_2_ exhibits high specific capacity, large working potential, low price, and slight toxicity, in comparison with other cathode materials (Pan et al., 2016; Xiong et al., 2019). The obtained flexible Zn-MnO_2_ battery based on the PAM/GO/EG gel electrolyte exhibits great electrochemical performances at temperatures from 20 to −20°C.

## Experimental

### Preparation of PAM/GO/EG Gel Electrolyte

GO was synthesized according to our previous report (Zhou W. et al., 2019). Firstly, 0.5 g EG was added to 30 mL of 1 mg/mL GO dispersion and stirred for 30 min. Then, 3 g acrylamide (AM) monomer was added to the above solution and stirred for 1 h, followed by ultrasonication for 15 min. Afterward, the resultant dispersion was added by 30 mg K_2_S_2_O_8_ (KPS) and stirred for 30 min. Subsequently, 4 mg N,N′-Methylenebisacrylamide (BIS) was added. After stirring for 30 min, the dispersion was poured into a mold. After polymerization at 60°C for 3 h and soaking into a mixed solution of 2 M ZnSO_4_ and 0.2 M MnSO_4_ in EG and water (the volumetric ratio of EG to water is 3: 7) for 24 h, the PAM/GO/EG gel electrolyte was finally obtained. For comparison, the PAM gel electrolyte without GO and EG was prepared by a similar method.

### Preparation of Flower-Like δ-MnO_2_

In a typical synthesis, 50 mL of 0.1 M MnSO_4_·H_2_O aqueous solution was dropwise added into 50 mL of 0.1 M KPS aqueous solution under vigorous stirring. After all the MnSO_4_·H_2_O aqueous solution was added, the mixed solution was stirred vigorously for another 1 h. Next, 30 mL of 1.2 M NaOH aqueous solution was added into the abovementioned mixed solution dropwise with vigorous stirring for 1 h, followed by aging for 1.5 h in the air. The obtained precipitate was washed with deionized (DI) water for 3 times, and then freeze-dried to obtain flower-like δ-MnO_2_.

### Characterization and Electrochemical Measurements

The morphology, structure, and chemical composition of samples were analyzed by JEOL JSM-7600F field-emission scanning electron microscope (FE-SEM), JEOL JEM-2100F transmission electron microscope (TEM), Rigaku Ultima IV powder X-ray diffractometer (XRD) with Cu Ka radiation source (λ = 1.5406 Å), and SANS UTM2502 universal mechanical testing machine. For the preparation of cathodes, δ-MnO_2_ cathode material was mixed with Super-P carbon black and polyvinylidene fluoride (PVDF) with a weight ratio of 7: 2: 1 in N-methyl pyrrolidone (NMP). The obtained slurry was coated onto a Ti foil and put into an oven at 80°C overnight. The mass loading of δ-MnO_2_ on the Ti foil is around 4 mg cm^−2^. The flexible Zn-MnO_2_ batteries were assembled by sandwiching the gel electrolyte between the δ-MnO_2_ cathode and the Zn foil anode. Galvanostatic charge/discharge (GCD) measurements were carried out on a LAND CT2001A battery testing system. Cyclic voltammetry (CV) and electrochemical impedance spectroscopy (EIS) measurements were performed on a Biologic VSP-300 electrochemical workstation. All the electrochemical measurements were conducted in a Shanghai Yiheng high-low temperature chamber.

## Results and Discussions

**Figure 1:**
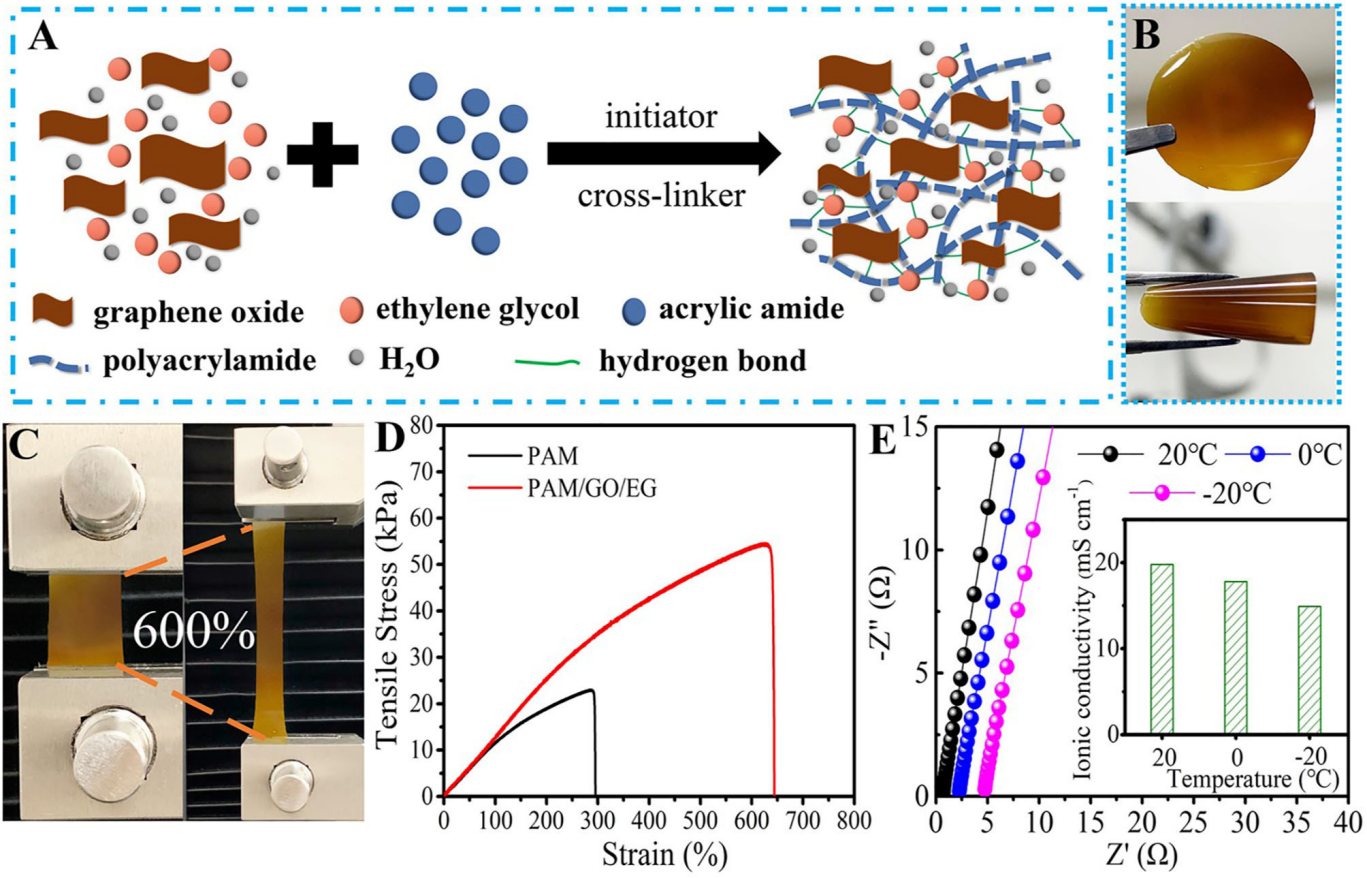
**(A)** The schematic representation of PAM/GO/EG gel. Photographs of PAM/GO/EG gel **(B)** at normal and bending states and **(C)** at normal and stretching states. **(D)** Tensile stress–strain curves of PAM and PAM/GO/EG gels. **(E)** Nyquist plots and the ionic conductivity values of PAM/GO/EG gel electrolyte at different temperatures.

The synthesis procedure of PAM/GO/EG gel is illustrated in Figure 1A. AM, GO, and EG were mixed in an aqueous system, followed by the successive addition of KPS and BIS under stirring. Then, the mixture was transferred to tailor-made molds and kept at 60°C for 3 h to obtain the PAM/GO/EG gel, whose photographs are shown in Figure 1B. The gel is observed to be semitransparent and homogeneous and possesses yellow color due to the presence of GO. Besides, the gel also shows great bendability. Thanks to the abundant hydrogen bonds among PAM, GO, and EG, a three-dimensional (3D) network is formed within the gel, as shown in Figure 1A. In particular, the inorganic GO with robust structure and high specific surface area can help construct a 3D macroporous network that facilitates ionic transport. It can also act as an effective reinforcer, benefiting from abundant oxygen-containing groups that can form abundant hydrogen bonds with hydrophilic polymer chains (Huang et al., 2016; Shi et al., 2016; Yang et al., 2019). The tensile stress–strain measurements were performed on cuboid hydrogel samples (~35 mm in length, ~20 mm in width, and ~1.5 mm in thickness) using a universal mechanical testing machine at a crosshead speed of 10 mm min^−1^. As presented in Figures 1C,D, the PAM/GO/EG gel possesses a tensile strength of 54.3 kPa and a fracture elongation of ~644%. In contrast, these values of PAM gel are much smaller. Furthermore, the PAM/GO/EG gel electrolyte owns high ionic conductivity values, which were determined from the EIS measurements over a frequency from 100 kHz to 0.01 Hz. According to the Nyquist plots in Figure 1E, the ionic conductivity values of gel electrolytes can be calculated. The calculation details can be found in our previous reports (Chen et al., 2020; Zhou et al., 2020). At a temperature of 20°C, the PAM/GO/EG gel electrolyte demonstrates a high ionic conductivity of 19.8 mS cm^−1^, which drops to 17.9 and 14.9 mS cm^−1^ when the temperature declines to 0 and −20°C, respectively. Such high ionic conductivity retentions at low temperatures are derived from the synergistic effect of EG and GO. To the best of our knowledge, the highest ionic conductivity of PAM-based gel electrolytes for AZIBs at −20°C is 14.6 mS cm^−1^ (Mo et al., 2019). Impressively, the PAM/GO/EG in this work offers a higher value, manifesting great applicability in low-temperature environments, while conventional aqueous electrolytes would fail at subzero temperatures.

**Figure 2:**
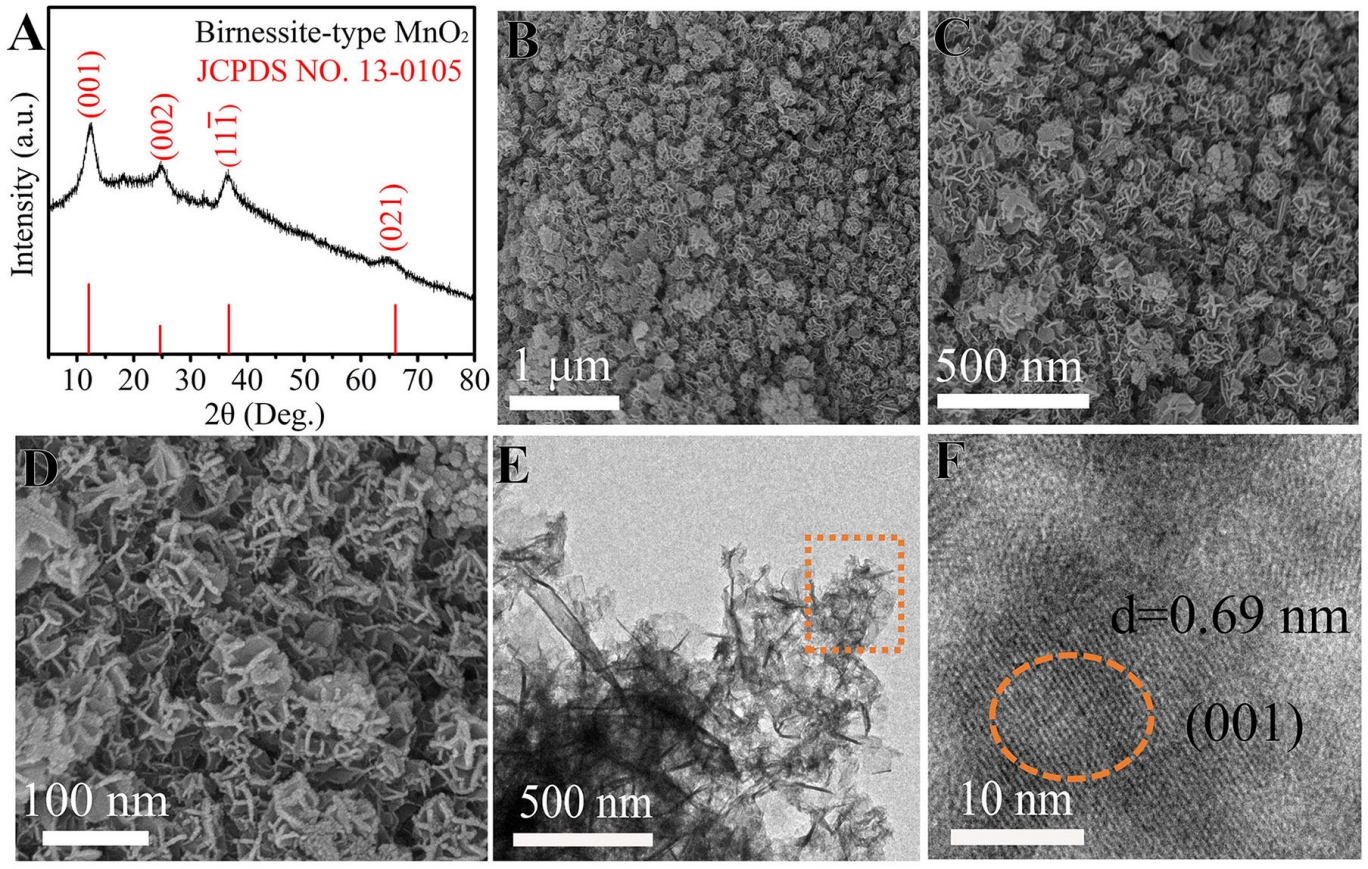
**(A)** XRD pattern, **(B–D)** SEM images, and **(E,F)** TEM images of δ-MnO_2_.

Up to now, a variety of cathode materials for AZIBs have been reported, mainly including Mn-based oxides (Huang J. H. et al., 2018; Liu et al., 2019; Guo et al., 2020), V-based oxides (Tang F. J. et al., 2019; Zhou W. J. et al., 2019), Mo-based oxides (Kim et al., 2017), Prussian blue analogs (Trocoli and La Mantia, 2015), and organic and polymer compounds (Li B. Q. et al., 2018). Among them, MnO_2_ might be the most promising one, primarily in terms of specific capacity and working potential (Ming et al., 2019; Zeng et al., 2019). Consequently, we used MnO_2_ as the cathode material to construct Zn-MnO_2_ batteries to study the benefits of PAM/GO/EG gel electrolyte. As shown in Figure 2A, all the four XRD peaks of the cathode material synthesized in this study at 12.1°, 24.7°, 36.8°, and 66.1° can be indexed to (001), (002), (11–1), and (021) planes of birnessite MnO_2_ (JCPDS 13-0105) (Alfaruqi et al., 2015). The broadness of these peaks implies small crystalline sizes, in favor of electrochemical reactions. The obtained δ-MnO_2_ possess a 2D-layered structure with a large interlayer distance, which can easily accommodate foreign cations such as Zn^2+^ ions, promoting the charge storage process. The morphology and structure of δ-MnO_2_ were investigated by SEM and TEM. Figures 2B–E show that δ-MnO_2_ possesses a homogenous flower-like morphology, and these flowers are around 100–150 nm in diameter and composed of several curved nanosheets stacked in a cross way. The HRTEM image in Figure 2F reveals a large lattice fringe of 0.69 nm, corresponding to the (001) plane of birnessite MnO_2_.

**Figure 3:**
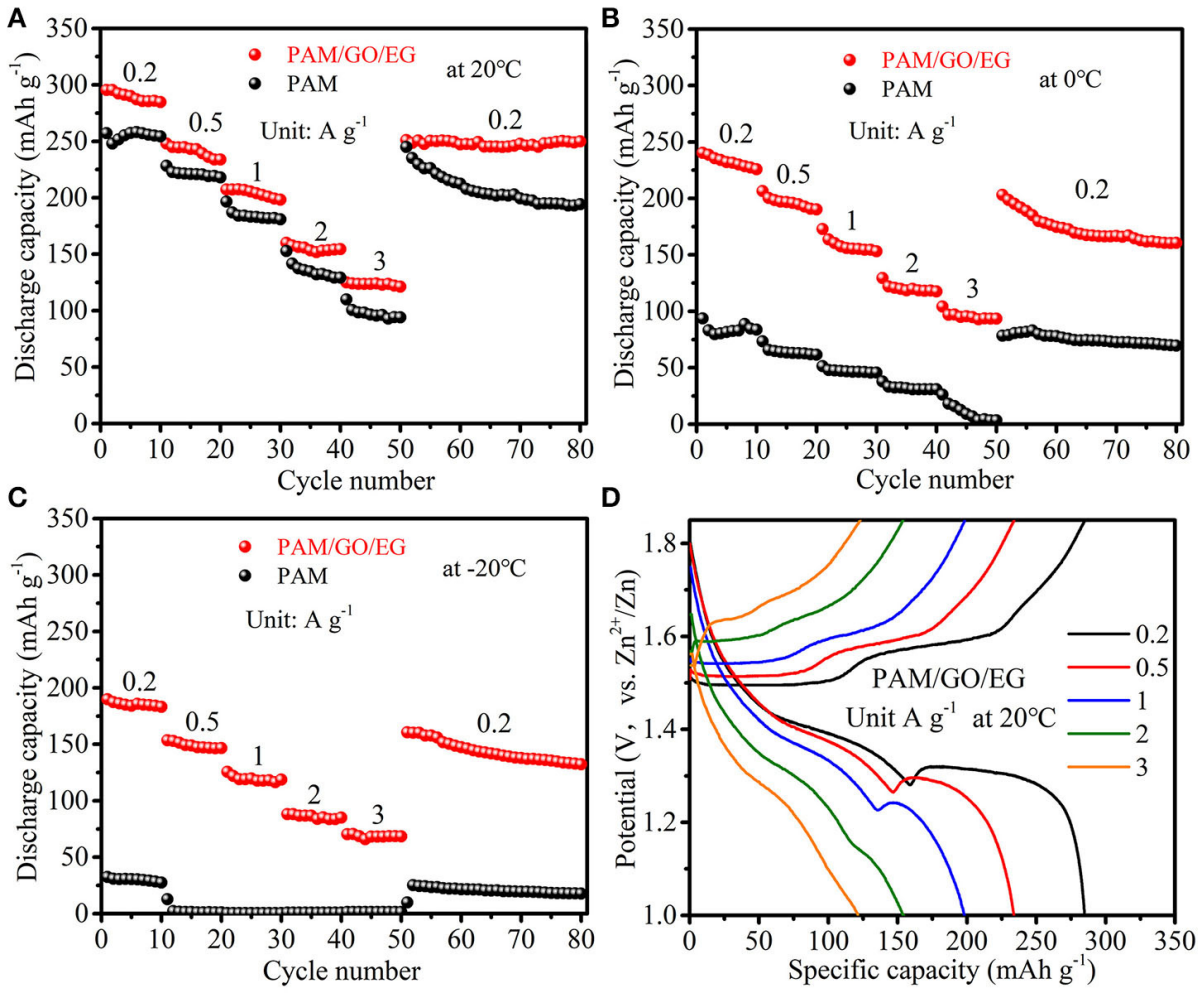
Rate performances of Zn-MnO_2_ batteries with PAM/GO/EG and PAM gel electrolytes: **(A)** at 20°C, **(B)** at 0°C, and **(C)** at −20°C. **(D)** GCD curves of Zn-MnO_2_ battery with PAM/GO/EG at 20°C.

The PAM/GO/EG gel electrolyte was combined with δ-MnO_2_ cathode material to assemble Zn-MnO_2_ batteries. As shown in Figure 3A, the Zn-MnO_2_ battery with PAM/GO/EG delivers a large discharge capacity of 284.8 mAh g^−1^ at 0.2 A g^−1^ (10th cycle) when the temperature is 20 °C. As the current density is raised to 0.5, 1, 2, and 3 A g^−1^ progressively, the discharge capacity drops to 233.8, 198.2, 154.4, and 121.3 mAh g^−1^ at the end of each current density, respectively. When the current density returns back to 0.2 A g^−1^, a large capacity of 249.9 mAh g^−1^ can be retained at the 80th cycle. The GCD curves at different current densities are presented in Figure 3D. Thanks to the high ionic conductivity of PAM/GO/EG and great electrochemical activity of δ-MnO_2_, the electrochemical polarization is not very severe at high current densities. For comparison, neat PAM gel electrolyte was also used. As can be seen from Figure 3A, the discharge capacities of Zn-MnO_2_ battery with PAM are much lower than that with PAM/GO/EG at all the current densities. The difference is much more prominent at lower temperatures (Figures 3B,C). At 0°C, the Zn-MnO_2_ battery with PAM/GO/EG gives 225.8, 190.3, 153.3, 117.6, and 93.5 mAh g^−1^ at 0.2, 0.5, 1, 2, and 3 A g^−1^, respectively. When the temperature is further decreased to −20°C, 183.2, 146.7, 118.7, 85.1, and 68.5 mAh g^−1^ can still be obtained, respectively. Such performances at low temperatures are much superior to that of previously reported anti-freezing and flexible EES devices (Mo et al., 2019; Wang et al., 2019). In sharp contrast, the discharging capacities of Zn-MnO_2_ battery with PAM are below 100 mAh g^−1^ at 0°C, and this battery almost cannot work at −20°C. Due to the high water content and lack of anti-freezing agent, the neat PAM gel electrolyte would (partially) freeze when the temperature drops to 0°C or below, thus hindering the transportation of electrolyte ions. Besides, the Zn-MnO_2_ battery with PAM/GO/EG can also deliver high energy densities of 390.2, 311.2, and 243.1 Wh kg^−1^ at 20, 0, and −20°C, respectively.

**Figure 4:**
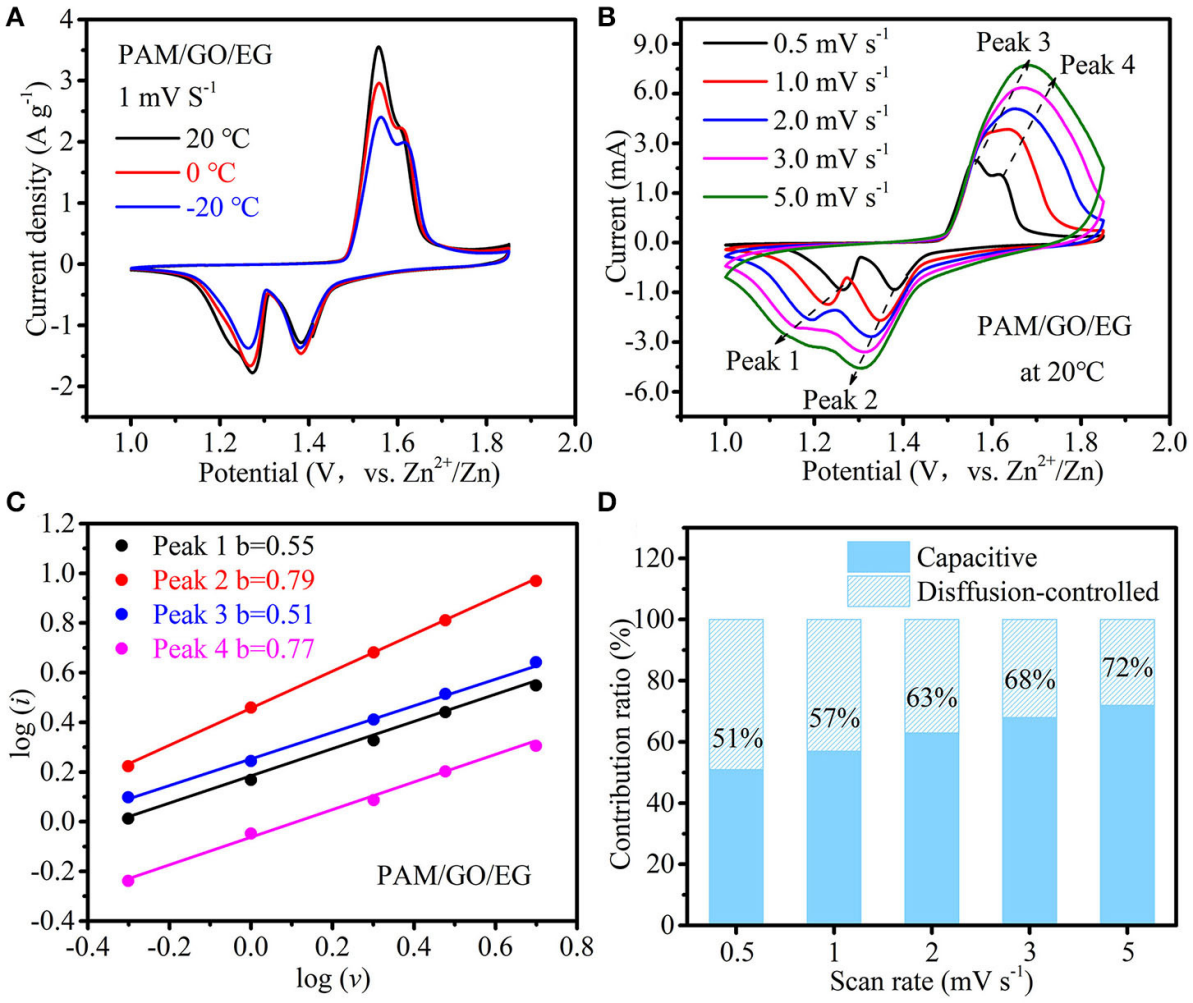
**(A)** CV curves at 1 mV s^−1^ at different temperatures, **(B)** CV curves at different scan rates at 20°C, **(C)** the linear fitting of log (*i*) *vs*. log (*v*) for four redox CV peaks, and **(D)** contribution ratios of diffusion-controlled and capacitive charge storage with respect to the scan rate of Zn-MnO_2_ battery with the PAM/GO/EG gel electrolyte.

The temperature-dependent electrochemical properties of Zn-MnO_2_ battery with PAM/GO/EG were first examined by CV at 1 mV s^−1^ (Figure 4A). When the temperature was gradually decreased from 20 to −20°C at longer cycles, the CV peaks remain well-defined, the peak positions change very slightly, and the decline in peak current is not severe, indicative of great anti-freezing ability. CV measurements at scan rates from 0.5 to 5 mV s^−1^ were conducted at 20°C to analyze the electrochemical kinetics of Zn-MnO_2_ battery with PAM/GO/EG, as shown in Figure 4B. With the increase in scan rate, the anodic peaks move to the positive position, while the cathodic peaks move to the opposite direction at the same time. This phenomenon is due to the increased electrochemical polarization at a higher scan rate. The general relationship between peak current (*i*) and scan rate (ν) can be described by the following equation (Augustyn et al., 2013):

(1) $$\text{i}=\text{a}\nu^{\text{b}}$$

A larger *b* value would reflect higher contribution of capacitive charge storage. Based on the linear fitting between log (*i*) and log (ν) (Figure 4C), the *b* values associated with four redox peaks are calculated to be 0.55, 0.79, 0.51, and 0.71, suggesting that capacitive charge storage plays an important role in this battery. Furthermore, the capacitive contribution can be separated using the following equation (Ming et al., 2018; Chen L. N. et al., 2019):

(2) $$\text{i}\;(\text{V})={\text{k}}_{1}\nu +{\text{k}}_{2}\nu^{1/2}$$

where *k*_1_ν and *k*_2_ν^1/2^ represent the capacitive and diffusion-controlled parts of the current response (*i*(*V*)), respectively. As illustrated in Figure 4D, the capacitive contribution increases from 51 to 72% when the scan rate increases from 0.5 to 5 mV s^−1^. That is, the capacitive charge storage accounts for more than half of the total capacity, and its proportion rises with the increase in scan rate, implying fast charge storage kinetics, which results from high ionic conductivity of PAM/GO/EG, as well as nanostructure and fast Zn^2+^ ion diffusion within the δ-MnO_2_ cathode material.

**Figure 5:**
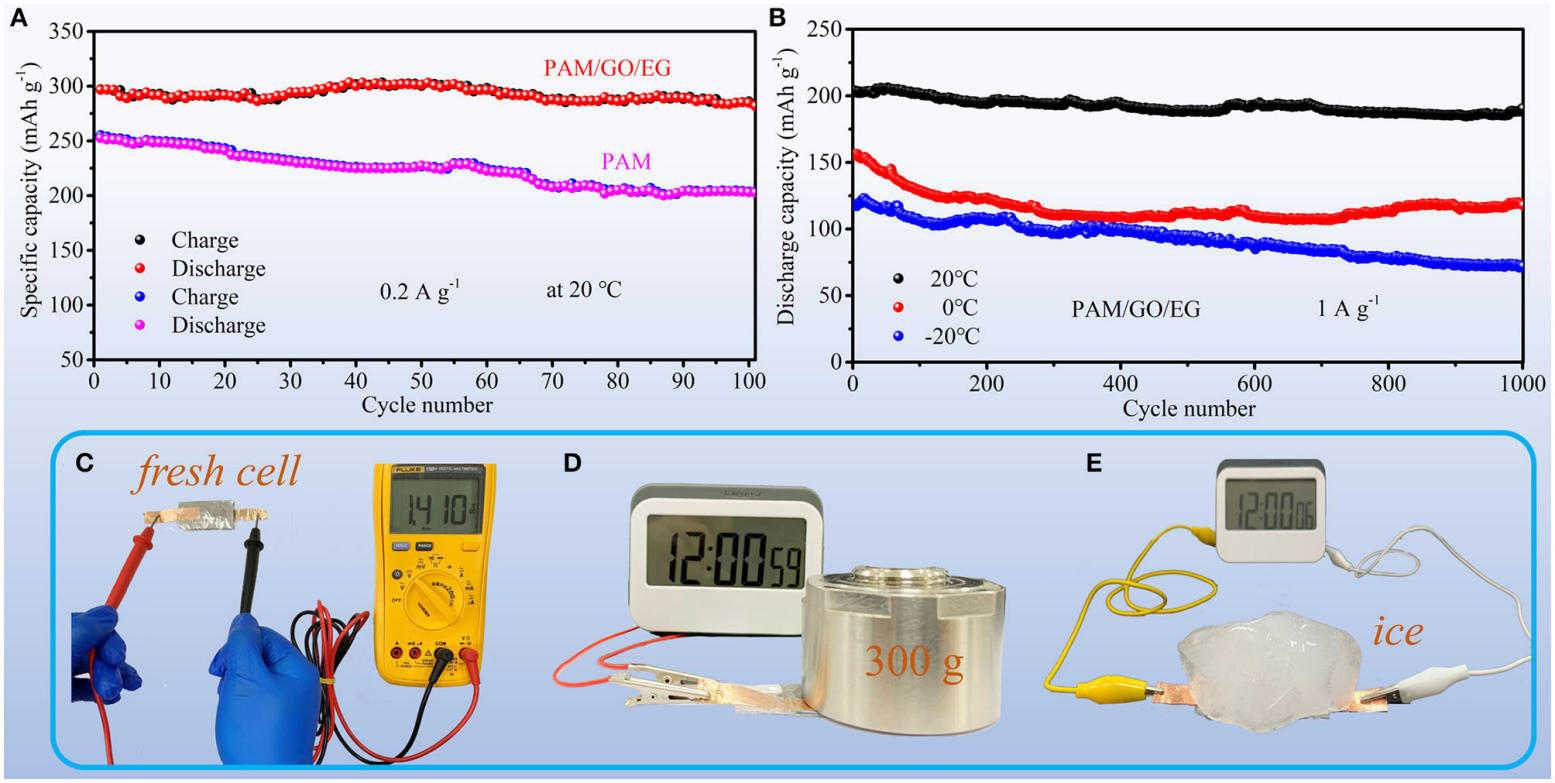
Cycling performances of Zn-MnO_2_ batteries: **(A)** with PAM/GO/EG and PAM gel electrolytes at 0.2 A g^−1^ at 20°C; **(B)** with PAM/GO/EG at 1 A g^−1^ at different temperatures. **(C)** The photograph of measuring the open-circuit voltage of a Zn-MnO_2_ battery with PAM/GO/EG. Demonstrations of powering an electric timer with a Zn-MnO_2_ battery with PAM/GO/EG **(D)** when the battery is compressed by a weight and **(E)** when the battery is compressed by a solid ice.

The cycling performances of the Zn-MnO_2_ batteries with different electrolytes at 20°C are compared in Figure 5A. The Zn-MnO_2_ battery with PAM/GO/EG exhibits great cycling stability. After 100 cycles at 0.2 A g^−1^, the battery can maintain 95.0% of its initial capacity. On the contrary, the capacity retention of Zn-MnO_2_ battery with PAM is 80.2% after 100 cycles. Figure 5B shows long-term cycling performances of Zn-MnO_2_ battery with PAM/GO/EG at different temperatures. After 1,000 cycles, the capacity retentions are 93.7, 75.6, and 61.0% at 20, 0, and −20°C, respectively. These results indicate that the introduction of GO and EG into PAM gel electrolyte can not only improve cyclability but also endow the Zn-MnO_2_ battery with great anti-freezing ability. In order to further reflect the practical application prospects of Zn-MnO_2_ battery with PAM/GO/EG, some demonstrations are shown in Figures 5C–E. The freshly assembled battery offers an open-circuit voltage of 1.41 V (Figure 5C). Although this value is much lower than LIBs with organic electrolytes (Han et al., 2018), it is higher than previously reported AZIBs (Guo et al., 2019; Zhang et al., 2019). The much lower voltage is reasonable, since severe water splitting would occur if another cathode material with a higher working potential is used. During the daily usage of flexible energy storage devices, they would be frequently compressed. As demonstrated in Figures 5D,E and Supplementary Video 1, our battery works normally under compression even at a low temperature. Such good load-bearing and anti-freezing behaviors mainly derive from great properties of PAM/GO/EG gel electrolyte.

## Conclusion

With the fast development of wearable electronics and outstanding advantages of AZIBs, it is highly desirable to develop high-performance flexible AZIBs. Realization of gel electrolytes with high ionic conductivity is a key to the practical applications of flexible AZIBs. Meanwhile, currently reported gel electrolytes are usually confronted with poor freezing resistance and low mechanical strength. In this study, we have successfully developed a high-performance anti-freezing flexible Zn-MnO_2_ battery based on the PAM/GO/EG gel electrolyte. Thanks to the synergistic effect of GO and EG, the mechanical properties, ionic conductivity, and anti-freezing ability of the gel electrolyte can be significantly improved in comparison with the neat PAM gel electrolyte. As a result, the Zn-MnO_2_ battery with PAM/GO/EG can deliver high reversible specific capacities of 284.8, 225.8, and 183.2 mAh g^−1^ at 20, 0, and −20°C, respectively, at a current density of 0.2 Ag^−1^. The battery also shows great rate capability and good cyclability at different temperatures. This work provides a novel strategy for exploring low-cost anti-freezing gel electrolytes for flexible power supplies.

## Data Availability Statement

The original contributions presented in the study are included in the article/supplementary material, further inquiries can be directed to the corresponding author/s.

## Author Contributions

All authors extensively discussed the results, reviewed the manuscript, and approved the final version of the manuscript to be published.

## Conflict of Interest

The authors declare that the research was conducted in the absence of any commercial or financial relationships that could be construed as a potential conflict of interest.

## Supplementary Material

Supplementary Video 1 Demonstration of the Zn-MnO2 battery with PAM/GO/EG powering a small electric fan.
